# Supplementary material for: Genome-Wide Association Study of Brain Connectivity Changes for Alzheimer’s Disease
Source: Sci Rep. 2020 Jan 29;10:1433. doi: 10.1038/s41598-020-58291-1 (PMC6989662; doi:10.1038/s41598-020-58291-1)
Supplement: Supplementary file 1 — Supplementary Info. [file 41598_2020_58291_MOESM1_ESM.pdf]

Supplementary Materials for: Genome-Wide Association Study of Brain Connectivity Changes for Alzheimer's Disease

Samar S. M. Elsheikh, Emile R. Chimusa, Nicola J. Mulder and Alessandro Crimi

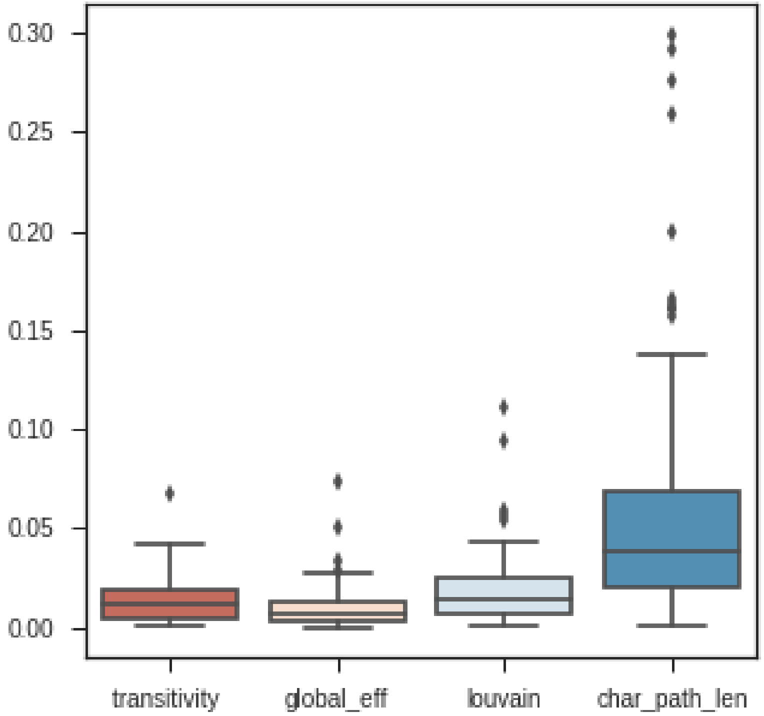

Figure S1. Distribution of global network metrics for controls, MCI and AD subjects, combined. Shortcuts stand for; Louvain: Louvain modularity, global_eff: global effeciency, and char_path_len: characteristic path length

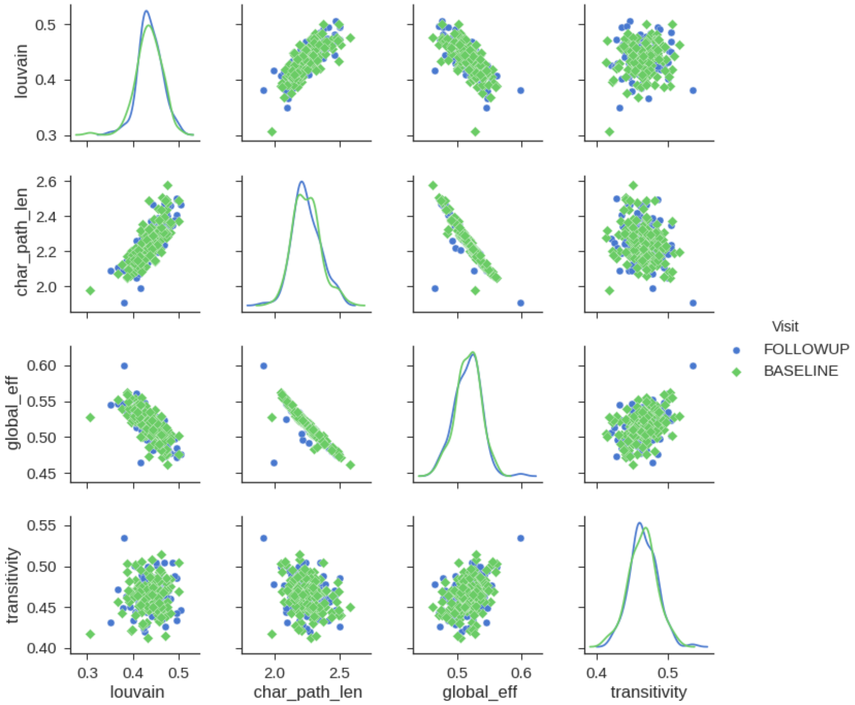

Figure S2. Global network metrics scatter plots: The sub-plots compare the four global network metrics before and after 12 months (baseline vs follow-up). Diagonal plots show the distribution of the actual metrics in the baseline and follow-up, while the remaining plots show the correlation between the metrics, for all participants.

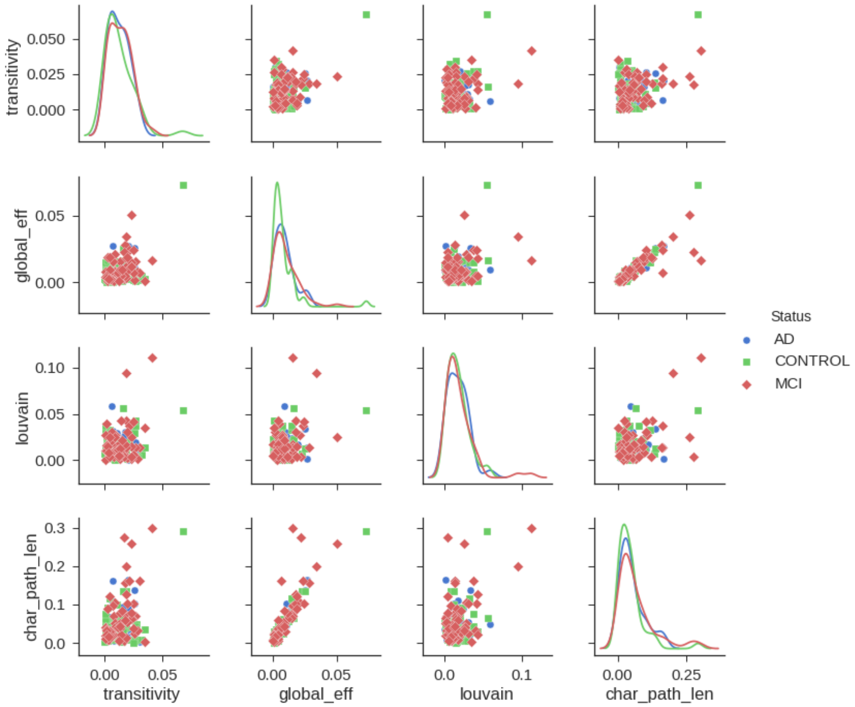

Figure S3. Global network metrics scatter plots: The sub-figures show the distribution of the absolute difference of the four network metrics (diagonal plots); as well as the pairwise correlation between them (remaining plots). Each plot compares AD, MCI and controls.

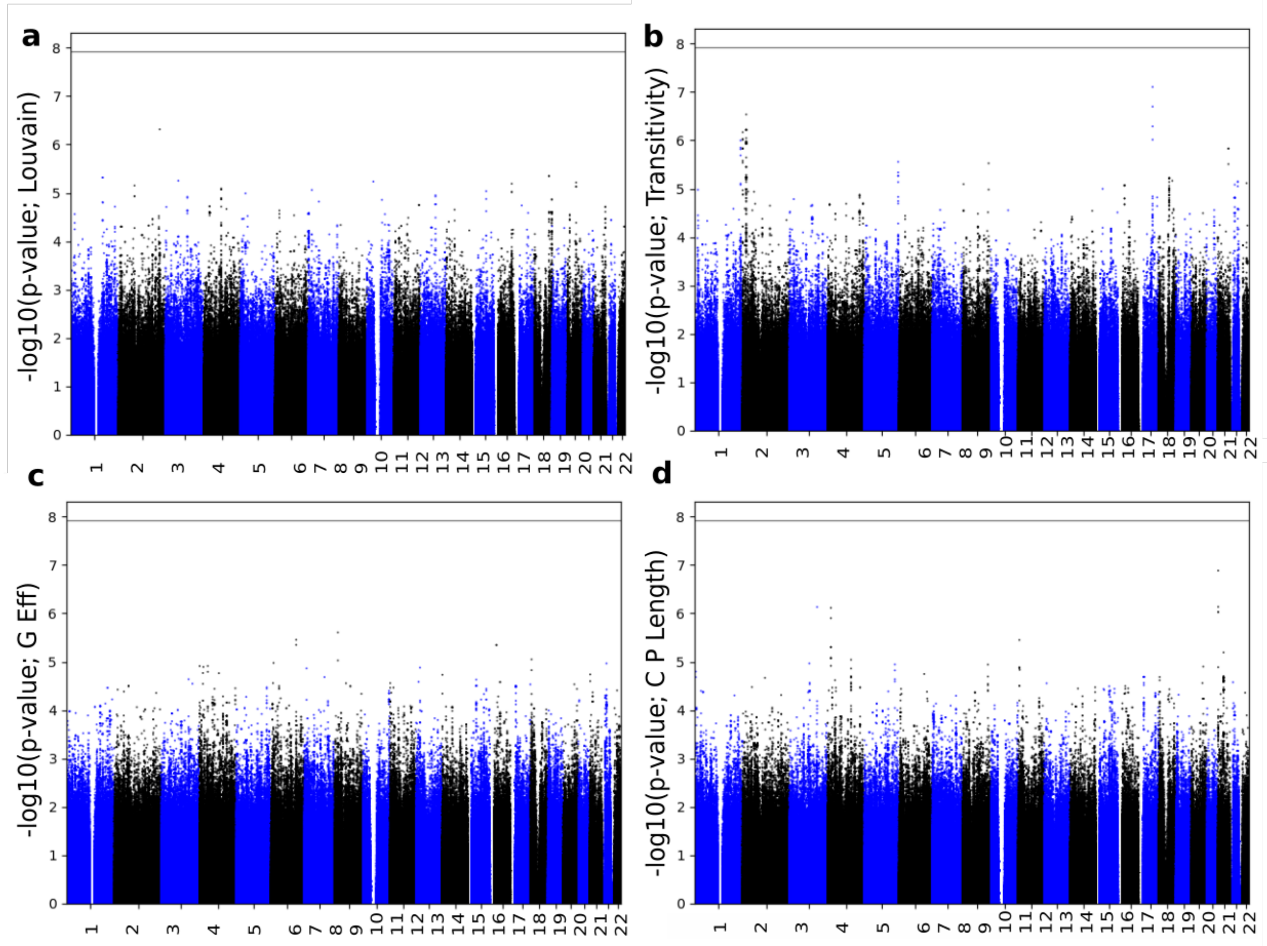

Figure S4. Manhattan plots of GWAS results for the change in Louvain modularity (a) and transitivity (b) global efficiency (c) and the change in characteristics path length (d) integration and segregation connectivity metrics.

Table S1. Louvain modularity GWAS results: Top 15 SNPs

**SNP**	**Dashed line is the 5% threshold**						
	Chr	BP	Eff/Alt	Type(R2)	Statistic	β	P
rs144596626	5	19473852	G/A	imputed (0.717)	6.32 (z)		2.68e-10
rs146631242	5	19396212	G/A	imputed (0.717)	6.32 (z)		2.68e-10
rs35942723	2	67943399	T/C	imputed (0.7)	5.43 (z)		5.5e-08
rs2460661	5	98903041	C/T	imputed (0.65)	5.3 (z)		1.14e-07
rs144454897	3	45220242	G/A	imputed (0.839)	-5.2 (z)		2.03e-07
rs12694279	2	213253406	C/A	gwas (1)	-5.992 (t)	-1.19	4.85e-07
rs146293495	11	87983529	C/T	imputed (0.782)	-5.01 (z)		5.54e-07
rs145955468	4	182593928	G/A	imputed (0.818)	4.88 (z)		1.07e-06
rs185097390	4	182653779	G/A	imputed (0.818)	4.88 (z)		1.07e-06
rs149021889	5	19168938	T/G	imputed (0.603)	4.82 (z)		1.42e-06
rs189745822	7	130949241	C/A	imputed (0.805)	-4.8 (z)		1.56e-06
rs193071172	7	130946162	G/A	imputed (0.805)	-4.8 (z)		1.56e-06
rs150749209	7	42493974	T/C	imputed (0.714)	-4.66 (z)		3.12e-06
rs189358029	17	43702340	C/T	imputed (0.437)	4.62 (z)		3.87e-06
rs8053032	16	75181579	G/C	gwas (1)	-5.199 (t)	-0.9548	4.5e-06

Table S2. Transitivity GWAS results: Top 15 SNPs

SNP	Chr	BP	Eff/Alt	Type(R2)	Statistic	β	P
	**Dashed line is the 5% threshold**						
rs4617614	11	55537046	C/T	imputed (0.717)	5.54 (z)		3.11e-08
rs144573130	1	65415375	C/T	imputed (0.82)	5.53 (z)		3.27e-08
rs111650215	15	78828083	A/G	imputed (0.661)	-5.43 (z)		5.69e-08
rs112671439	15	78819074	T/C	imputed (0.661)	-5.43 (z)		5.69e-08
rs113809575	15	78833209	G/A	imputed (0.661)	-5.43 (z)		5.69e-08
rs113882269	15	78833286	A/G	imputed (0.661)	-5.43 (z)		5.69e-08
rs11912587	22	38371933	A/C	imputed (0.738)	5.37 (z)		7.81e-08
rs4459504	15	71883930	G/A	gwas (1)	6.192(t)	0.8938	7.83e-08
rs144750443	5	89847749	C/T	imputed (0.745)	-5.2 (z)		1.96e-07
rs3923493	15	71866632	T/C	gwas (1)	5.973 (t)	0.9766	2e-07
rs2518679	14	31252534	T/C	imputed (0.679)	5.18 (z)		2.21e-07
rs61156477	14	31266735	T/C	imputed (0.679)	5.18 (z)		2.21e-07
rs77762911	14	31255027	T/G	imputed (0.679)	5.18 (z)		2.21e-07
rs8018229	14	31261372	G/A	imputed (0.679)	5.18 (z)		2.21e-07
rs147801202	2	19102920	A/ATG	gwas (1)	-5.939 (t)	-1.049	2.91e-07

Table S3. Global efficiency GWAS results: Top 15 SNPs

SNP	Chr	BP	Eff/Alt	Type(R2)	Statistic	β	P
	**Dashed line is the 5% threshold**						
rs112039371	4	126730783	T/C	imputed (0.775)	-6.53 (z)		6.48e-11
rs114045002	4	126746229	C/A	imputed (0.775)	-6.53 (z)		6.48e-11
rs76699517	4	126741800	T/C	imputed (0.775)	-6.53 (z)		6.48e-11
rs78276525	4	126732179	G/T	imputed (0.775)	-6.53 (z)		6.48e-11
rs78538713	4	126742275	T/C	imputed (0.775)	-6.53 (z)		6.48e-11
rs78570105	4	126749594	G/A	imputed (0.775)	-6.53 (z)		6.48e-11
rs7657714	4	126735951	A/C	imputed (0.792)	-6.12 (z)		9.12e-10
rs113323321	4	80897619	C/T	imputed (0.743)	5.85 (z)		4.85e-09
rs192963808	12	102764731	A/G	imputed (0.694)	5.53 (z)		3.28e-08
rs146655189	12	102674455	C/T	imputed (0.717)	5.48 (z)		4.27e-08
rs148061827	10	109846291	G/T	imputed (0.678)	4.84 (z)		1.28e-06
rs2139572	12	102767660	C/T	imputed (0.657)	4.82 (z)		1.44e-06
rs149903755	14	100808726	A/G	imputed (0.804)	-4.77 (z)		1.86e-06
rs149119261	10	108038891	C/T	imputed (0.762)	4.73 (z)		2.3e-06
rs62497351	8	17086601	G/T	gwas (1)	5.278 (t)	1.402	2.48e-06

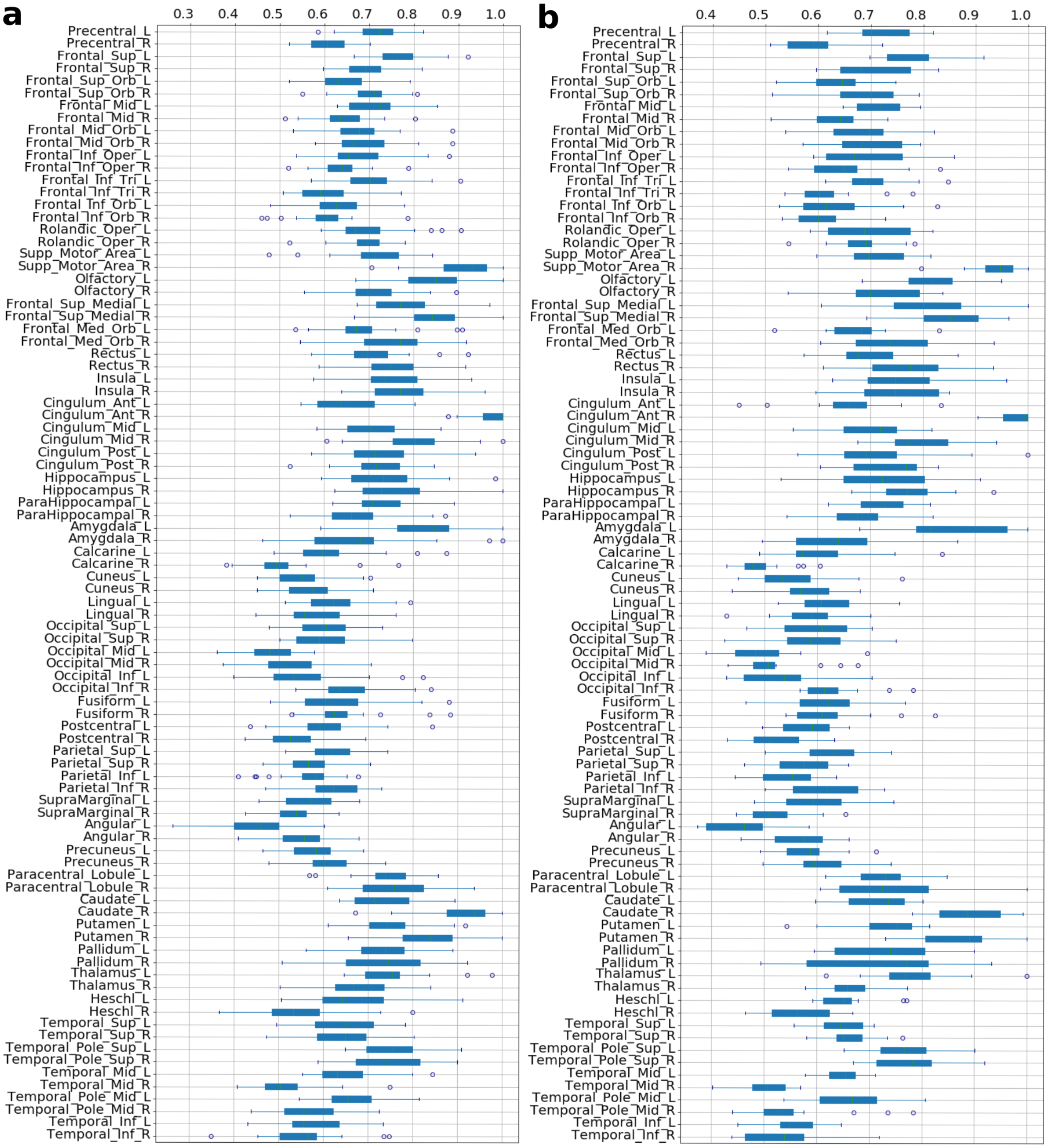

Figure S5. Boxplots of hubs degree centrality averaged according to the single ROI of the AAL atlas. On the left (a) are the values for the AD subjects at baseline, and (b) on the right are the values for the AD subjects at follow-up.

Table S4. Charactristic path length GWAS results: Top 15 SNPs

**SNP**	**Dashed line is the 5% threshold**						
	Chr	BP	Eff/Alt	Type(R2)	Statistic	β	P
rs112039371	4	126730783	T/C	imputed (0.775)	-6.73 (z)		1.7e-11
rs114045002	4	126746229	C/A	imputed (0.775)	-6.73 (z)		1.7e-11
rs76699517	4	126741800	T/C	imputed (0.775)	-6.73 (z)		1.7e-11
rs78276525	4	126732179	G/T	imputed (0.775)	-6.73 (z)		1.7e-11
rs78538713	4	126742275	T/C	imputed (0.775)	-6.73 (z)		1.7e-11
rs78570105	4	126749594	G/A	imputed (0.775)	-6.73 (z)		1.7e-11
rs7657714	4	126735951	A/C	imputed (0.792)	-6.39 (z)		1.64e-10
rs10113946	9	131534333	C/T	imputed (0.725)	-5.36 (z)		8.29e-08
rs11560592	9	131532694	C/T	imputed (0.725)	-5.36 (z)		8.29e-08
rs28521006	9	131534909	G/A	imputed (0.725)	-5.36 (z)		8.29e-08
rs35354551	20	2112390	C/T	gwas (1)	-6.089 (t)	-1.107	1.31e-07
rs113323321	4	80897619	C/T	imputed (0.743)	5.22 (z)		1.83e-07
rs10122433	9	131577388	A/C	imputed (0.753)	-5.18 (z)		2.25e-07
rs12236573	9	131555366	G/A	imputed (0.715)	-5.03 (z)		5.01e-07
rs10115869	9	131652502	G/A	imputed (0.735)	-5.03 (z)		5.03e-07

Table S5. Top 20 gene sets (pathways) results derived from GWAS summary statistics of global network metrics.

Gene Set	Results are sorted by p-value.		
	Chr	Pvalue	Metric
**REACTOME_BIOLOGICAL_OXIDATIONS**	10	2.91E-5	Louvain
**REACTOME_REGULATION_OF_ORNITHINE_DECARBOXYLASE_ODC**	15	5.2E-5	Transitivity
**KEGG_ALDOSTERONE_REGULATED_SODIUM_REABSORPTION**	12	6.6E-5	G Efficiency
**BIOCARTA_IL7_PATHWAY**	1	6.9E-5	Transitivity
**REACTOME_IL_6_SIGNALING**	1	7.6E-5	Transitivity
**KEGG_PATHWAYS_IN_CANCER**	12	1.05E-4	G Efficiency
**BIOCARTA_ERYTH_PATHWAY**	12	1.32E-4	G Efficiency
**BIOCARTA_BAD_PATHWAY**	12	1.38E-4	G Efficiency
**KEGG_PROGESTERONE_MEDIATED_OOCYTE_MATURATION**	12	1.38E-4	G Efficiency
**BIOCARTA_IL22BP_PATHWAY**	1	1.57E-4	Transitivity
**REACTOME_GENERIC_TRANSCRIPTION_PATHWAY**	2	2.03E-4	G Efficiency
**KEGG_PROSTATE_CANCER**	12	2.46E-4	G Efficiency
**REACTOME_ACTIVATION_OF_NMDA_RECEPTOR_ UPON_GLUTAMATE_BINDING_AND_POSTSYNAPTIC_EVENTS**	8	2.56E-4	Transitivity
**REACTOME_POST_NMDA_RECEPTOR_ACTIVATION_EVENTS**	8	2.7E-4	Transitivity
**KEGG_LYSOSOME**	11	3.8E-4	Louvain
**REACTOME_OLFACTORY_SIGNALING_PATHWAY**	11	4.08E-4	Transitivity
**REACTOME_ANTIVIRAL_MECHANISM_BY_IFN_STIMULATED_GENES**	1	4.17E-4	Transitivity
**REACTOME_REGULATION_OF_IFNA_SIGNALING**	1	4.27E-4	Transitivity
**BIOCARTA_IL2_PATHWAY**	1	4.29E-4	Transitivity
**KEGG_GLIOMA**	12	4.34E-4	G Efficiency
